# Supplementary material for: Multimodal mechanisms of human centriole engagement and disengagement
Source: EMBO J. 2025 Feb 4;44(5):1294–321. doi: 10.1038/s44318-024-00350-8 (PMC11876316; doi:10.1038/s44318-024-00350-8)
Supplement: Supplementary file 13 — Expanded View Figures [file 44318_2024_350_MOESM13_ESM.pdf]

Expanded View Figures

Figure EV1. Effect of HU and CDK2 inhibitor treatments on centriole and cell cycle.

(**A**) Representative immunofluorescence images of HeLa cells treated with HU or HU + CDK2 inhibitor. Scale bar: 5 µm. (**B**) Quantification of the p-Rb (Ser807/811) signal intensity in the nucleus from (**A**). Boxplot was employed to illustrate the data distribution. The central line represents the median, the box spans the interquartile range (IQR) from the 25th to the 75th percentile, and the whiskers extend to the smallest and largest data points within 1.5 times the IQR. *P* values were calculated by two-tailed unpaired Student's t-test ($P = 1.57E-08$). ***$P < 0.001$. (**C**) Quantification of the DNA content in HeLa cells treated as indicated, using flow cytometric analysis. (**D–F**) Scatter plot quantification of the length and the width of the daughter centrioles from Fig. 1. (**G**) Representative immunofluorescence images of HeLa cells in early S phase, late S phase, G2 phase, and mitosis. Scale bars: 5 µm, 1 µm. (**H**) Quantification of the frequency of cells with disengaged centrioles in G2 phase in (**G**). Error bars represent the mean ± s.d. of three biological independent experiments. *P* value was calculated by Dunnett's multiple comparisons test ($P = 1.24E-07$). ***$P < 0.001$. (**I**) Quantification of the frequency of cells with disengaged centrioles in mitosis in (**G**). Error bars represent the mean ± s.d. of three biological independent experiments. *P* value was calculated by Dunnett's multiple comparisons test ($P = 8.06E-10$). ***$P < 0.001$. Source data are available online for this figure.

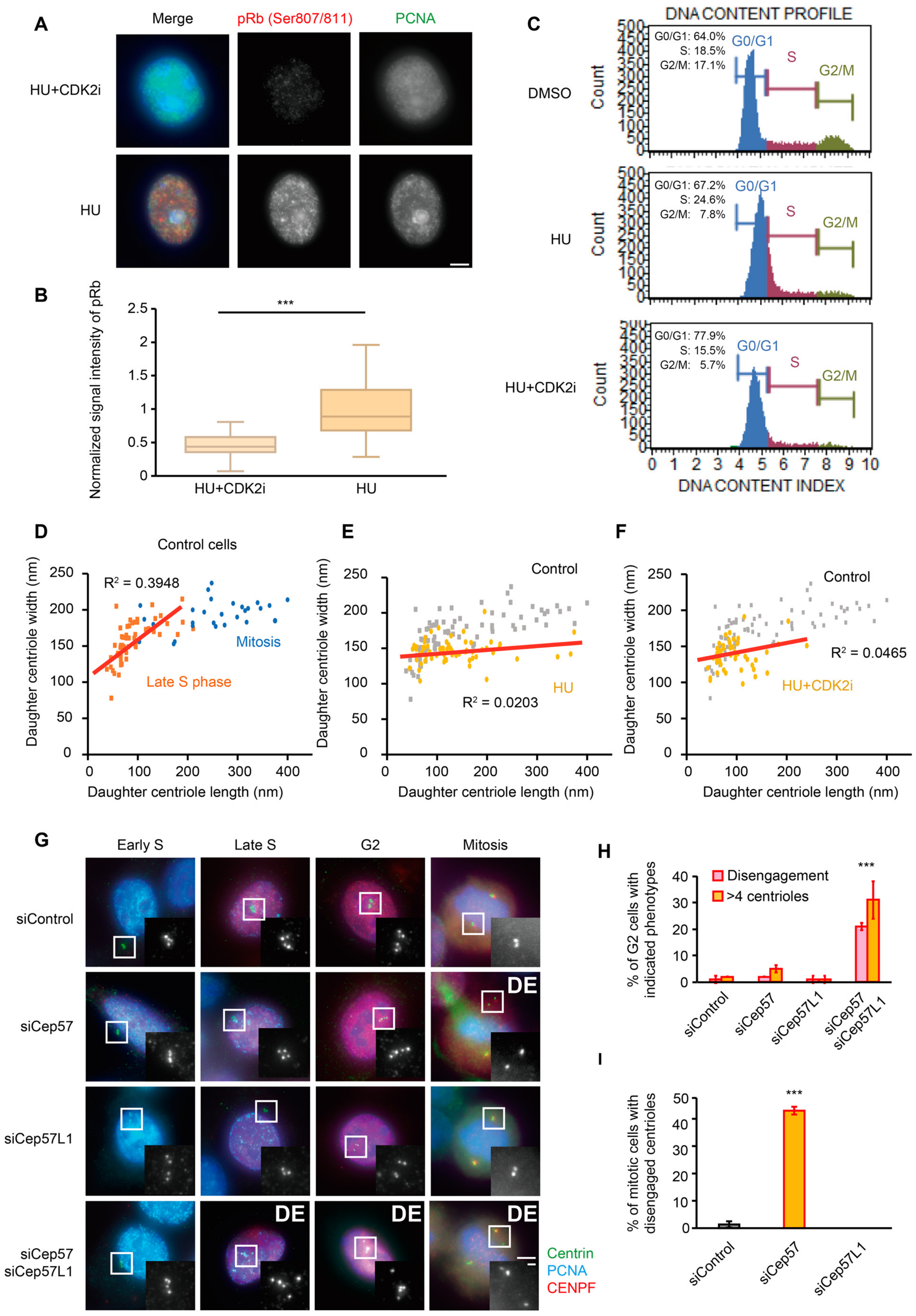
A
Merge
pRb (Ser807/811)
PCNA
HU+CDK2i
HU
B
Normalized signal intensity of pRb
***
HU+CDK2i
HU
C
DNA CONTENT PROFILE
DMSO
G0/G1: 64.0%
S: 18.5%
G2/M: 17.1%
HU
G0/G1: 67.2%
S: 24.6%
G2/M: 7.8%
HU+CDK2i
G0/G1: 77.9%
S: 15.5%
G2/M: 5.7%
Count
G0/G1
S
G2/M
DNA CONTENT INDEX
D
Control cells
R² = 0.3948
Mitosis
Late S phase
Daughter centriole width (nm)
Daughter centriole length (nm)
E
Control
HU
R² = 0.0203
F
Control
R² = 0.0465
HU+CDK2i
G
Early S
Late S
G2
Mitosis
siControl
siCep57
siCep57L1
siCep57 siCep57L1
DE
Centrin
PCNA
CENPF
H
% of G2 cells with indicated phenotypes
Disengagement
>4 centrioles
***
siControl
siCep57
siCep57L1
siCep57 siCep57L1
I
% of mitotic cells with disengaged centrioles
***
siControl
siCep57
siCep57L1

 © The Author(s)

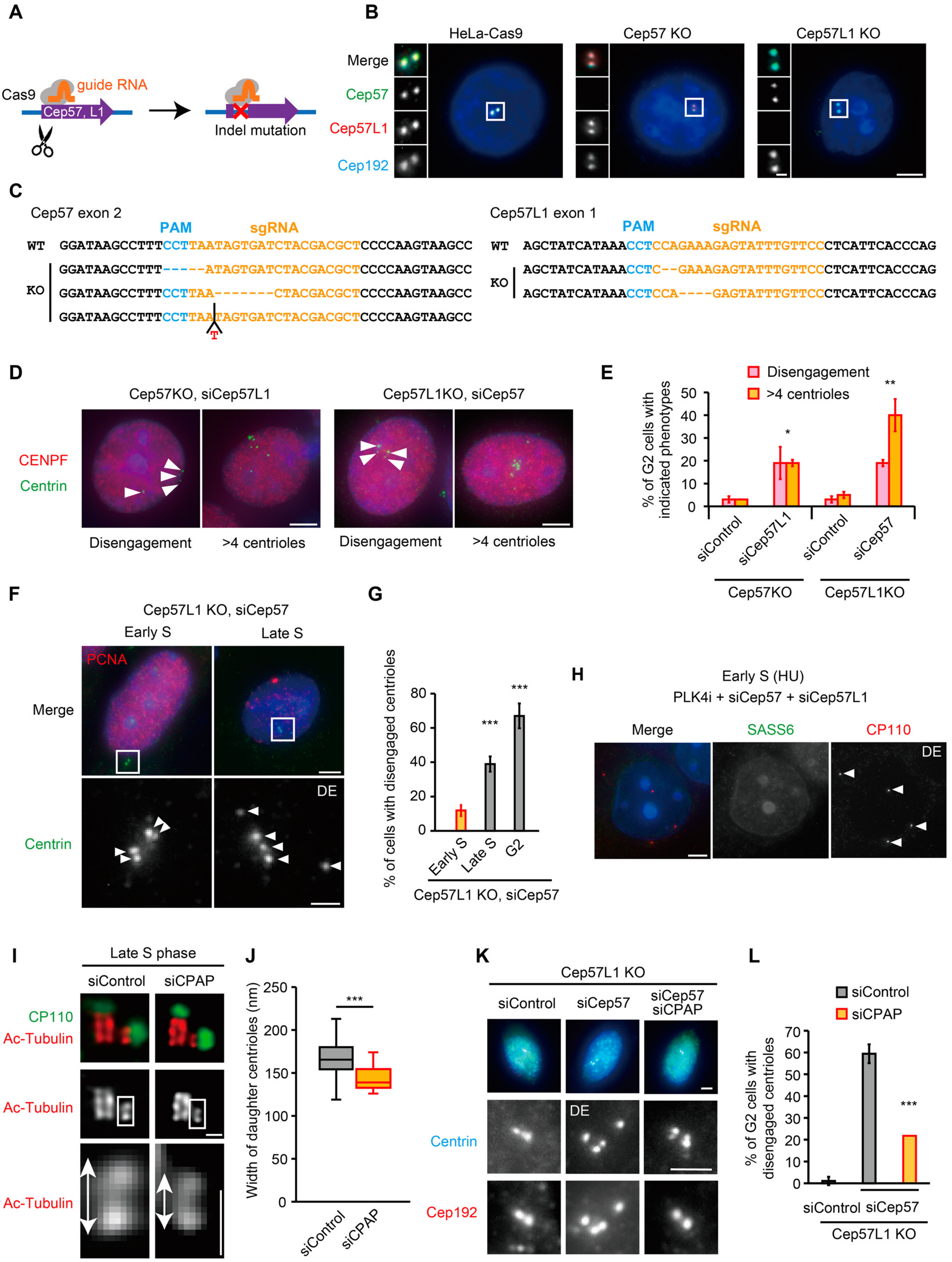
A
Cas9
guide RNA
Cep57, L1
Indel mutation
B
HeLa-Cas9
Cep57 KO
Cep57L1 KO
Merge
Cep57
Cep57L1
Cep192
C
Cep57 exon 2
PAM
sgRNA
WT GGATAAGCCTTTCCTTAATAGTGATCTACGACGCTCCCCAAGTAAGCC
KO
GGATAAGCCTTT-----ATAGTGATCTACGACGCTCCCCAAGTAAGCC
GGATAAGCCTTTCCTTAA-------CTACGACGCTCCCCAAGTAAGCC
GGATAAGCCTTTCCTTAATAGTGATCTACGACGCTCCCCAAGTAAGCC
T
Cep57L1 exon 1
PAM
sgRNA
WT AGCTATCATAAACCTCCAGAAAGAGTATTTGTTCCCTCATTCACCCAG
KO
AGCTATCATAAACCTC--GAAAGAGTATTTGTTCCCTCATTCACCCAG
AGCTATCATAAACCTCCA----GAGTATTTGTTCCCTCATTCACCCAG
D
Cep57KO, siCep57L1
Cep57L1KO, siCep57
CENPF
Centrin
Disengagement
>4 centrioles
Disengagement
>4 centrioles
E
Disengagement
>4 centrioles
% of G2 cells with indicated phenotypes
50
40
30
20
10
0
*
**
siControl
siCep57L1
siControl
siCep57
Cep57KO
Cep57L1KO
F
Cep57L1 KO, siCep57
Early S
Late S
PCNA
Merge
Centrin
DE
G
% of cells with disengaged centrioles
80
60
40
20
0
***
***
Early S
Late S
G2
Cep57L1 KO, siCep57
H
Early S (HU)
PLK4i + siCep57 + siCep57L1
Merge
SASS6
CP110
DE
I
Late S phase
siControl
siCPAP
CP110
Ac-Tubulin
Ac-Tubulin
Ac-Tubulin
J
Width of daughter centrioles (nm)
250
200
150
100
50
0
***
siControl
siCPAP
K
Cep57L1 KO
siControl
siCep57
siCep57 siCPAP
Centrin
DE
Cep192
L
siControl
siCPAP
% of G2 cells with disengaged centrioles
70
60
50
40
30
20
10
0
***
siControl
siCep57
Cep57L1 KO

© The Author(s)

Figure EV2. Confirmation of the centriole engagement by Cep57 and Cep57L1 in interphase using knockout cell lines.

(**A**) Schematic illustration for generating knockout (KO) cells using CRISPR-Cas9. (**B**) Immunofluorescence images of HeLa-Cas9, Cep57KO, and Cep57L1KO cells. Scale bars: 5 µm, 1 µm. (**C**) DNA sequences surrounding the CRISPR-targeted regions in the exons of the Cep57 and Cep57L1 genes. (**D**) Representative immunofluorescence images of HeLa-Cep57KO cells transfected with siCep57L1, and Cep57L1KO cells transfected with siCep57 in G2 phase. Scale bars: 5 µm. White arrowheads indicate the centrioles. (**E**) Quantification of the frequency of cells possessing four disengaged centrioles and more than four centrioles in G2 phase in (**D**). Error bars represent the mean ± s.d. of three biological independent experiments. P value was calculated by two-tailed unpaired Welch's t-test with Bonferroni correction (Cep57KO, $P = 0.0230$; Cep57L1KO, $P = 0.00146$). *$P < 0.05$; **$P < 0.01$. (**F**) Representative immunofluorescence images of HeLa-Cep57L1KO cells transfected with siCep57 in early and late S phase. Scale bars: 5 µm, 1 µm. White arrowheads indicate the centrioles. (**G**) Quantification of the frequency of the cells with disengaged centrioles in early S, late S, and G2 phase in (**F**). Error bars represent the mean ± s.d. of three biological independent experiments. P values were calculated by Dunnett's multiple comparisons test (Late S, $P = 0.000192$; G2, $P = 1.70E{-}06$). ***$P < 0.001$. (**H**) Representative immunofluorescence images of HeLa cells subjected to a cartwheel removal assay. HeLa cells were treated with HU and CDK2 inhibitor III (CDK2i) for 24 h, followed by treatment with a PLK4 inhibitor for another 24 h. Scale bar: 5 µm. White arrowheads indicate the centrioles. (**I**) Representative STED microscopy images of HeLa cells transfected with siControl or siCPAP. Double-headed arrows indicate the width of centrioles. Scale bars: 200 nm. (**J**) Quantification of the width of the daughter centrioles in (**I**). $n = 40$ centrioles pooled from 3 independent experiments. P value was calculated by two-tailed unpaired Welch's t-test ($P = 5.54E{-}07$). ***$P < 0.001$. (**K**) Representative immunofluorescence images of HeLa Cep57L1 KO cells transfected with siControl, siCep57, or siCep57 + siCPAP. Scale bars: 5 µm, 1 µm. (**L**) Quantification of the frequency of cells with disengaged centrioles in (**K**). Error bars represent the mean ± s.d. of three biological independent experiments. P value was calculated by two-tailed unpaired Welch's t-test ($P = 0.000419$). ***$P < 0.001$. Source data are available online for this figure.

 © The Author(s)

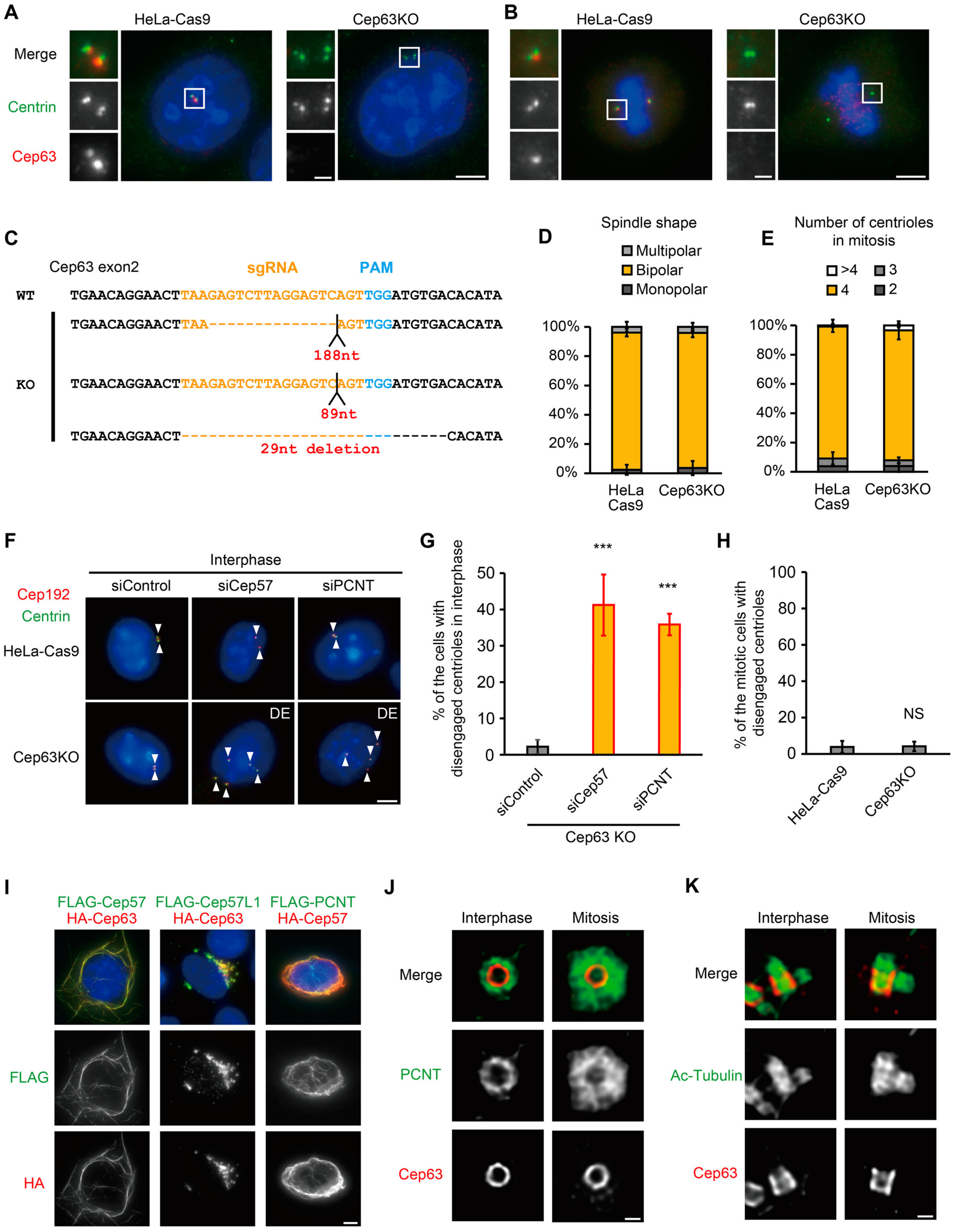
A
HeLa-Cas9
Cep63KO
Merge
Centrin
Cep63
B
HeLa-Cas9
Cep63KO
C
Cep63 exon2
sgRNA
PAM
WT TGAACAGGAACTTAAGAGTCTTAGGAGTCAGTTGGATGTGACACATA
KO
TGAACAGGAACTTAA---------------AGTTGGATGTGACACATA
188nt
TGAACAGGAACTTAAGAGTCTTAGGAGTCAGTTGGATGTGACACATA
89nt
TGAACAGGAACT------------------------------------CACATA
29nt deletion
D
Spindle shape
Multipolar
Bipolar
Monopolar
100%
80%
60%
40%
20%
0%
HeLa Cas9
Cep63KO
E
Number of centrioles in mitosis
>4
3
4
2
F
Interphase
siControl
siCep57
siPCNT
Cep192
Centrin
HeLa-Cas9
Cep63KO
DE
DE
G
% of the cells with disengaged centrioles in interphase
***
***
siControl
siCep57
siPCNT
Cep63 KO
H
% of the mitotic cells with disengaged centrioles
NS
HeLa-Cas9
Cep63KO
I
FLAG-Cep57 HA-Cep63
FLAG-Cep57L1 HA-Cep63
FLAG-PCNT HA-Cep57
FLAG
HA
J
Interphase
Mitosis
Merge
PCNT
Cep63
K
Interphase
Mitosis
Merge
Ac-Tubulin
Cep63

Figure EV3. Cep63 is involved in centriole engagement.

(**A**) Immunofluorescence images of HeLa-Cas9 and Cep63KO cells in interphase. Scale bars: 5 µm, 1 µm. (**B**) Immunofluorescence images of HeLa-Cas9 and Cep63KO cells in mitosis. Scale bars: 5 µm, 1 µm. (**C**) DNA sequences surrounding the CRISPR-targeted regions in the exons of the *Cep63* gene. (**D**) Quantification of the frequency of mitotic cells with the indicated spindle shape. Error bars represent the mean ± s.d. of three biological independent experiments. (**E**) Quantification of the frequency of mitotic cells with the indicated number of centrioles in (**B**). Error bars represent the mean ± s.d. of three biological independent experiments. (**F**) Representative immunofluorescence images of HeLa-Cas9 and Cep63KO cells transfected with siControl, siCep57 or siPCNT in interphase. Scale bar: 5 µm. White arrowheads indicate the centrioles. (**G**) Quantification of the frequency of cells with disengaged centrioles in (**F**). Error bars represent the mean ± s.d. of three biological independent experiments. P values were calculated by Dunnett's multiple comparisons test (siCep57: $P = 0.000234$, siPCNT: $P = 0.000496$). ***$P < 0.001$. (**H**) Quantification of the frequency of mitotic cells with disengaged centrioles. Error bars represent the mean ± s.d. of three biological independent experiments. P value was calculated by two-tailed unpaired Welch's t-test ($P = 0.912$). NS, not significant. (**I**) Immunofluorescence images of HeLa cells overexpressing Cep57, Cep57L1, Cep63, or PCNT after plasmid transfection. Scale bar: 5 µm. (**J**) STED microscopy image showing the top view of a mother centriole in G2 and mitosis. Scale bar: 200 nm. (**K**) STED microscopy image showing the side view of a mother centriole in G2 and mitosis. Scale bar: 200 nm. Source data are available online for this figure.

 © The Author(s)

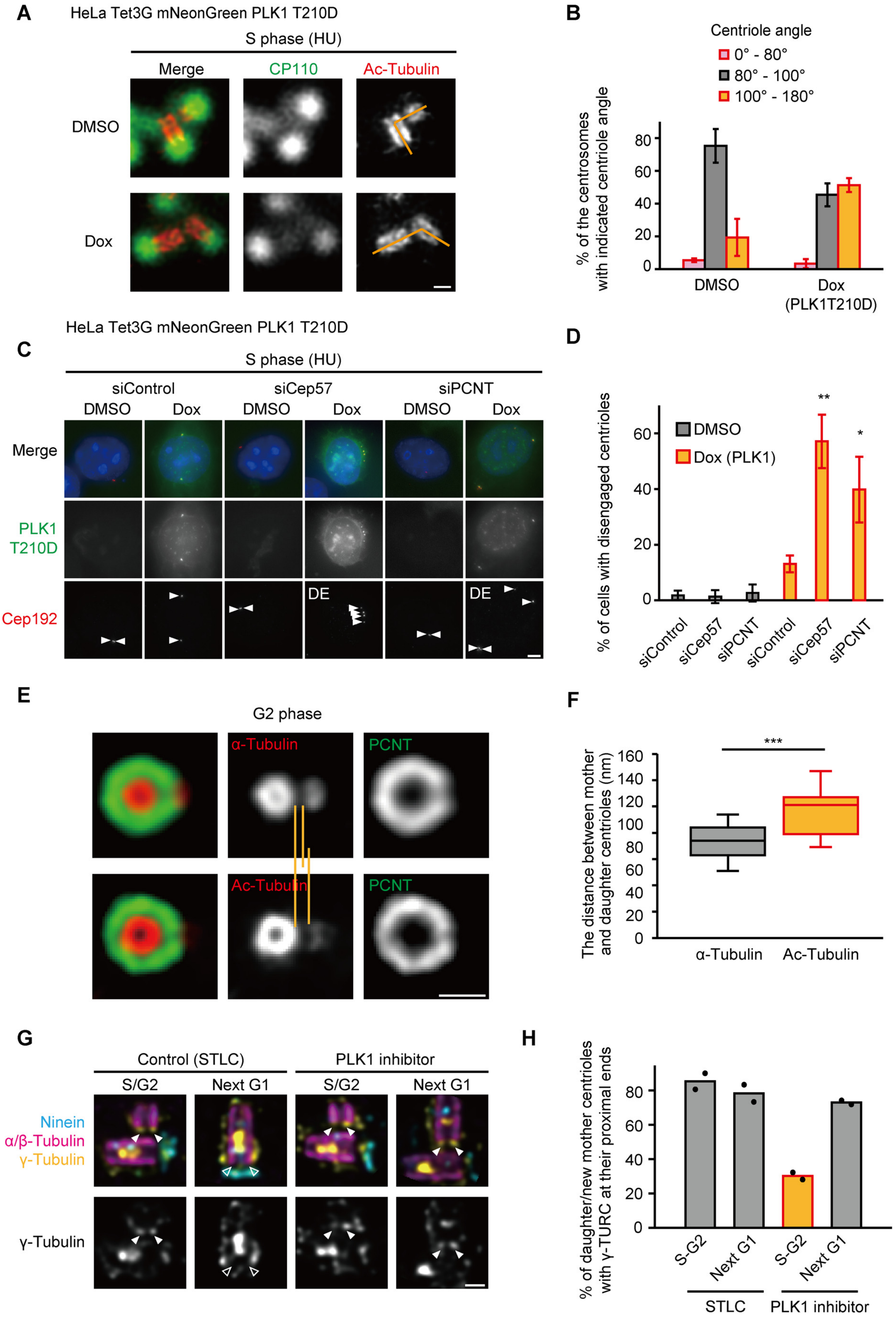
A
HeLa Tet3G mNeonGreen PLK1 T210D
S phase (HU)
Merge
CP110
Ac-Tubulin
DMSO
Dox
B
Centriole angle
0° - 80°
80° - 100°
100° - 180°
% of the centrosomes with indicated centriole angle
DMSO
Dox (PLK1T210D)
C
HeLa Tet3G mNeonGreen PLK1 T210D
S phase (HU)
siControl
siCep57
siPCNT
DMSO
Dox
Merge
PLK1 T210D
Cep192
DE
D
DMSO
Dox (PLK1)
% of cells with disengaged centrioles
**
*
E
G2 phase
α-Tubulin
PCNT
Ac-Tubulin
F
The distance between mother and daughter centrioles (nm)
***
α-Tubulin
Ac-Tubulin
G
Control (STLC)
PLK1 inhibitor
S/G2
Next G1
Ninein
α/β-Tubulin
γ-Tubulin
H
% of daughter/new mother centrioles with γ-TURC at their proximal ends
S-G2
Next G1
STLC
PLK1 inhibitor

Figure EV4. PLK1 overexpression changes the configuration and the mechanism of centriole engagement.

(**A**) Representative STED microscopy images of HeLa-tet3G-PLK1T210D cells treated with DMSO or Doxycycline in HU-arrested S phase. Scale bar: 200 nm. (**B**) Quantification of the frequency of cells with indicated centriole angle in (**A**). Error bars represent the mean ± s.d. of three biological independent experiments. (**C**) Representative immunofluorescence images of HeLa-tet3G-PLK1T210D cells treated with DMSO or Doxycycline in HU-arrested S phase. Scale bar: 5 μm. White arrowheads indicate the centrioles. (**D**) Quantification of the frequency of cells with disengaged centrioles in (**C**). Error bars represent the mean ± s.d. of three biological independent experiments. *P* values were calculated by Dunnett's multiple comparisons test (HU + Dox + siCep57: $P = 0.00490$, siPCNT: $P = 0.0447$). **P* < 0.05; ***P* < 0.01. (**E**) Representative STED microscopy images of centrioles in G2 phase immunostained with anti-PCNT and anti-α-tubulin or anti-acetylated tubulin antibodies. Scale bar: 200 nm. (**F**) Quantification of the distance between the mother centriole wall and the proximal end of the daughter centriole in (**E**). Boxplot was employed to illustrate the data distribution. The central line represents the median, the box spans the interquartile range (IQR) from the 25th to the 75th percentile, and the whiskers extend to the smallest and largest data points within 1.5 times the IQR. *P* value was calculated by two-tailed unpaired Student's t-test ($P = 0.000329$). ****P* < 0.001. (**G**) Representative U-ExM images of daughter and new mother centrioles in HeLa cells treated with STLC or the PLK1 inhibitor BI2536. New mother centrioles were obtained from cells forced into G1 by treatment with the prometaphase-arresting drugs STLC or BI2536 for 3 h, followed by the addition of the CDK1 inhibitor RO3306 (10 μM) for 3 h. New mother centrioles are identified by the absence of ODF2 signal at the distal part. Filled and open arrowheads indicate the proximal end of daughter/new mother centrioles with and without γ-tubulin signals, respectively. Scale bar: 200 nm. (**H**) Quantification of the frequency of the daughter or new mother centrioles with γ-tubulin signal at its proximal end in (**G**). Two biologically independent experiments, 30 cells each. Details of the quantification method are provided in the Methods section. Source data are available online for this figure.

 © The Author(s)

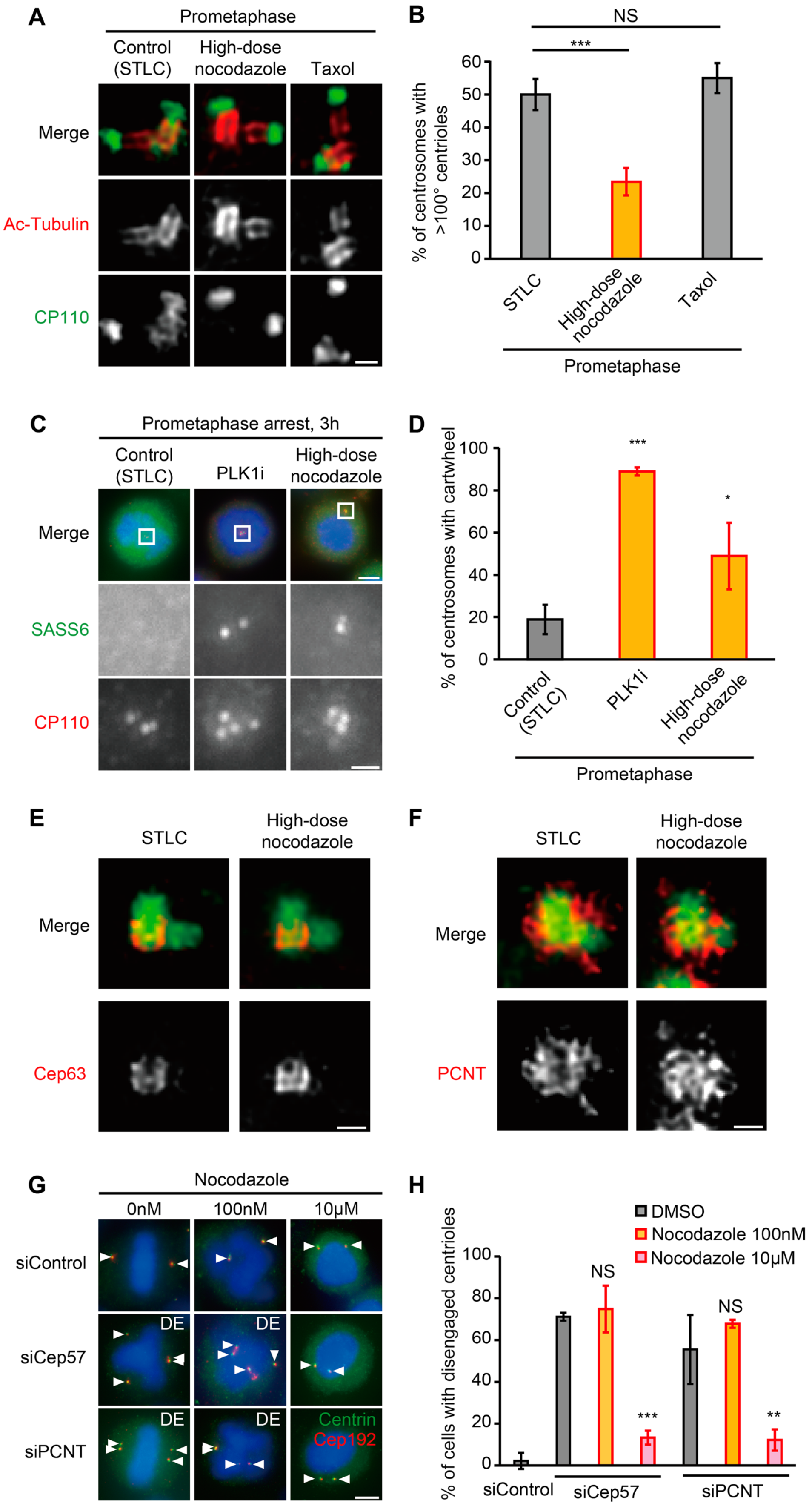
A
Prometaphase
Control (STLC)
High-dose nocodazole
Taxol
Merge
Ac-Tubulin
CP110
B
NS
***
% of centrosomes with >100° centrioles
60
50
40
30
20
10
0
STLC
High-dose nocodazole
Taxol
Prometaphase
C
Prometaphase arrest, 3h
Control (STLC)
PLK1i
High-dose nocodazole
Merge
SASS6
CP110
D
% of centrosomes with cartwheel
100
80
60
40
20
0
***
*
Control (STLC)
PLK1i
High-dose nocodazole
Prometaphase
E
STLC
High-dose nocodazole
Merge
Cep63
F
STLC
High-dose nocodazole
Merge
PCNT
G
Nocodazole
0nM
100nM
10μM
siControl
siCep57
siPCNT
DE
Centrin
Cep192
H
% of cells with disengaged centrioles
DMSO
Nocodazole 100nM
Nocodazole 10μM
100
80
60
40
20
0
NS
NS
***
**
siControl
siCep57
siPCNT

Figure EV5. The proximal end of the daughter centriole is depolymerized in mitosis.

(**A**) Representative STED microscopy images of centrioles in mitosis treated with DMSO, high-dose nocodazole (10 μM), or taxol for 3 h. Scale bar: 200 nm. (**B**) Quantification of the frequency of the centrosomes, in which the centrioles are connected at an obtuse angle in (**A**). Error bars represent the mean ± s.d. of three biological independent experiments. *P* values were calculated by Dunnett's multiple comparisons test (Nocodazole: $P = 0.000458$, Taxol: $P = 0.291$). NS, not significant; ***$P < 0.001$. (**C**) Representative immunofluorescence images of HeLa cells in mitosis treated with STLC, PLK1 inhibitor, or high-dose nocodazole for 3 h. Scale bar: 5 μm, 1 μm. (**D**) Quantification of the frequency of cells with cartwheel-retaining centrosomes in (**C**). Error bars represent the mean ± s.d. of three biological independent experiments. *P* values were calculated by Dunnett's multiple comparisons test (PLK1i: $P = 0.00029$, Nocodazole: $P = 0.0185$). *$P < 0.05$; ***$P < 0.001$. (**E**, **F**) Representative STED microscopy images of centrioles in HeLa cells. Scale bars: 200 nm. (**G**) Representative immunofluorescence images of HeLa cells in mitosis transfected with siControl, siCep57, or siPCNT, under treatment with different doses of nocodazole. Scale bar: 5 μm. White arrowheads indicate the centrioles. (**H**) Quantification of the frequency of cells with disengaged centrioles in mitosis in (**G**). Error bars represent the mean ± s.d. of three biological independent experiments. *P* values were calculated by Dunnett's multiple comparisons test (siCep57 100 nM nocodazole: $P = 0.739$, 10 μM nocodazole: $P = 0.000163$; siPCNT 100 nM nocodazole: $P = 0.299$, 10 μM nocodazole: $P = 0.00297$). NS, not significant; **$P < 0.01$; ***$P < 0.001$. Source data are available online for this figure.

© The Author(s)
